# A single-center, assessor-blinded, randomized controlled clinical trial to test the safety and efficacy of a novel brain-computer interface controlled functional electrical stimulation (BCI-FES) intervention for gait rehabilitation in the chronic stroke population

**DOI:** 10.1186/s12883-024-03710-3

**Published:** 2024-06-13

**Authors:** Piyashi Biswas, Lucy Dodakian, Po T. Wang, Christopher A. Johnson, Jill See, Vicky Chan, Cathy Chou, Wendy Lazouras, Alison L. McKenzie, David J. Reinkensmeyer, Danh V. Nguyen, Steven C. Cramer, An H. Do, Zoran Nenadic

**Affiliations:** 1 Department of Biomedical Engineering, University of California, Irvine, USA; 2 Department of Rehabilitation Services, University of California at Irvine Medical Center, Orange, USA; 3 Department of Epidemiology and Biostatistics, University of California, Irvine, USA; 4 Department of Neurology, University of California, Irvine, USA; 5 Department of Physical Therapy, Chapman University, Orange, USA; 6 Department of Anatomy and Neurobiology, University of California, Irvine, USA; 7 Department of Mechanical and Aerospace Engineering, University of California, Irvine, USA; 8 Department of General Internal Medicine, University of California, Irvine, USA; 9 Department of Neurology, University of California, Los Angeles, USA; 10 California Rehabilitation Institute, Los Angeles, USA; 11 Department of Electrical Engineering and Computer Science, University of California, Irvine, USA

**Keywords:** Stroke, Brain-computer interface, Electroencephalography, Functional electrical stimulation, Lower extremity rehabilitation, Gait velocity, Brain plasticity, Motor learning, Neurorehabilitation

## Abstract

**Background:**

In the United States, there are over seven million stroke survivors, with many facing gait impairments due to foot drop. This restricts their community ambulation and hinders functional independence, leading to several long-term health complications. Despite the best available physical therapy, gait function is incompletely recovered, and this occurs mainly during the acute phase post-stroke. Therapeutic options are limited currently. Novel therapies based on neurobiological principles have the potential to lead to long-term functional improvements. The Brain-Computer Interface (BCI) controlled Functional Electrical Stimulation (FES) system is one such strategy. It is based on Hebbian principles and has shown promise in early feasibility studies. The current study describes the BCI-FES clinical trial, which examines the safety and efficacy of this system, compared to conventional physical therapy (PT), to improve gait velocity for those with chronic gait impairment post-stroke. The trial also aims to find other secondary factors that may impact or accompany these improvements and establish the potential of Hebbian-based rehabilitation therapies.

**Methods:**

This Phase II clinical trial is a two-arm, randomized, controlled, longitudinal study with 66 stroke participants in the chronic (> 6 months) stage of gait impairment. The participants undergo either BCI-FES paired with PT or dose-matched PT sessions (three times weekly for four weeks). The primary outcome is gait velocity (10-meter walk test), and secondary outcomes include gait endurance, range of motion, strength, sensation, quality of life, and neurophysiological biomarkers. These measures are acquired longitudinally.

**Discussion:**

BCI-FES holds promise for gait velocity improvements in stroke patients. This clinical trial will evaluate the safety and efficacy of BCI-FES therapy when compared to dose-matched conventional therapy. The success of this trial will inform the potential utility of a Phase III efficacy trial.

**Trial registration:**

The trial was registered as ”BCI-FES Therapy for Stroke Rehabilitation” on February 19, 2020, at clinicaltrials.gov with the identifier NCT04279067.

## Background

Stroke is the most common neurological injury and is one of the leading causes of disability in the United States [1]. Over 795,000 new stroke cases occur every year [1], bringing a total census of chronic stroke survivors to *>* 7,000,000. Despite the best available physical therapy (PT), 30-60% of stroke survivors experience gait impairments [1–3]. Foot drop (FD) [4, 5]—the inability to actively dorsiflex the ankle during the swing phase of gait, is one of the most significant contributors to gait challenges. Those with FD develop compensatory movement patterns that significantly impact their gait velocity, limit their functional mobility, and make them reliant on orthotic and assistive devices [6]. This population is at a much higher risk of falls [7–10], which can cause further health complications [11, 12].

Poor mobility is of particular importance amongst post-stroke impairments because it limits participation in daily activities and hinders the social reintegration of this population [13–15]. Therefore, gait restoration remains a top rehabilitation priority amongst stroke survivors [16–18]. From a public health standpoint, these adverse outcomes lead to increased healthcare, higher caregiving burden, and greater lost productivity costs. This public health burden will be exacerbated as the aging population grows and acute stroke survival rates keep improving [19].

Despite decades of research dedicated to post-stroke rehabilitation, the options for alleviating chronic stroke-related walking disability remain limited. For example, clinicians rely on assistive devices like walkers, canes, and ankle-foot orthoses (AFO) to mitigate gait impairments. However, these devices are conspicuous, can cause discomfort, and their benefits mostly disappear upon removal. Approaches emphasizing intense activity are promising but at an early stage [20]. Hence, novel approaches that exceed the benefits of conventional PT and can potentially provide long-lasting functional gains are being investigated. Of these, the most notable examples include robotic devices [21] and body-weight-supported treadmill rehabilitation methods [22]. However, these interventions have not conclusively proven their effectiveness as superior to conventional therapy [2]. Functional electrical stimulation (FES) is another commonly used approach to mitigate post-stroke gait deficits. While primarily used in conjunction with AFOs to manage FD [23], repeated FES use may have a temporary “carryover” effect [24, 25]. However, there are contradictory reports in randomized controlled trials (RCTs) on the therapeutic effect of FES as a standalone therapy [23, 26–28].

Therapeutic gains in motor function generally require favorable forms of motor system plasticity. Examples include paired associative stimulation (PAS), whereby repetitive cortical and peripheral nerve stimulations are delivered in a precise temporal sequence [29]. PAS is thought to elicit Hebbian plasticity by coincident activation of the neurons within the primary motor cortex (M1) [30]. While PAS has shown promising preliminary results in stroke rehabilitation [31–33], these findings have not been confirmed in RCTs. Another stoke rehabilitation approach thought to harness neuroplasticity is electromyogram (EMG)-driven FES [34]. However, Phase I/II clinical trials showed that EMG-driven FES provided no benefits beyond conventional therapy, including FES alone [35–38].

Brain-computer interfaces (BCIs) are systems that perform real-time analysis of brain signals, e.g., electroencephalogram (EEG), and translate these into control commands for assistive devices [39]. When integrated with FES systems, BCIs could be used to deliver a novel form of post-stroke rehabilitation therapy. Early feasibility studies of this concept in the upper [40–43] and lower [44–46] extremity rehabilitation suggest that BCI-FES systems are safe and can improve post-stroke motor function, presumably by Hebbian plasticity.

We previously developed an EEG-based BCI-FES system that targets deficits in foot dorsiflexion [44, 47] and demonstrated its safety in a small (*n* = 9) Phase I trial with chronic stroke survivors with FD [45]. Specifically, after undergoing 12 one-hour sessions of BCI-FES therapy over four weeks, no subjects had safety concerns, including the absence of a decrement in any outcome measure, suggesting that the BCI-FES therapy is safe. Additionally, six out of nine subjects exhibited a detectable increase in either gait velocity and/or six-minute walk distance (6MWD). Moreover, five of these subjects exhibited an increase in their EEG *µ*- and *β*-band event-related synchronization/desynchronization, suggesting the emergence of a neuroplastic process underlying gait velocity increases.

In summary, these preliminary findings suggest that BCI-FES therapy for FD due to stroke is safe and potentially effective. Based on this, we concluded that a Phase II, two-arm RCT is necessary to formally ascertain the efficacy of this therapy in chronic stroke survivors with FD, results of which could inform further development of this approach. This RCT is outlined below.

## Aims and hypotheses

This article outlines the design of a Phase II clinical trial that investigates the safety and potential efficacy of BCI-FES in rehabilitating FD and resulting gait impairment due to chronic stroke. Specifically, we hypothesize that this intervention will result in greater improvement in gait velocity (primary outcome) of chronic stroke survivors with FD when compared to dose-matched conventional PT (standard of care). This hypothesis is motivated by the premise that BCI-FES therapy facilitates a coincident activation of M1 (detected by BCI) and *α*-motor neurons in spinal gray matter (antidromically activated via FES). Therefore, BCI-FES intervention may strengthen the connection between the brain and spinal motor pools through Hebbian plasticity and ultimately lead to lasting gains in gait function.

Additionally, we hypothesize that specific behavioral and physiological measures will predict an individual’s responsiveness to the BCI-FES therapy. This hypothesis is predicated on a model whereby key factors at baseline, such as gait velocity, dorsiflexion function, tibialis anterior (TA) muscle activity, and EEG sensorimotor rhythm biomarkers during attempted dorsiflexion, are needed to derive benefit from BCI-FES therapy and therefore affect the efficacy of this intervention.

The improvements associated with this therapy, along with the predictor factors, will be quantified by functional and neurophysiological assessments carried out at multiple time points before, during, and after the intervention. This study will help us determine the safety and efficacy of BCI-FES gait rehabilitation.

## Methods/design

### Trial design

This study is designed as a two-arm, parallel-group, assessor-blinded, Phase II superiority clinical trial of BCI-FES rehabilitation therapy where 66 participants with FD and gait impairment at the chronic stage of stroke are randomized into BCI-FES (experimental) group or dose-matched PT (control) group. The experimental group receives BCI-FES dorsiflexion therapy paired with conventional PT, while the control arm receives dose- and intensity-matched conventional PT. Enrollment, intervention, and assessments take place in a single-center clinical research setting (Institute for Clinical and Translational Science–ICTS) at the University of California Irvine (UCI), Irvine, California, USA. This study is reported following the SPIRIT guidelines (see Supplementary file 1).

All human subject procedures conducted during this trial are carried out in compliance with federal and institutional ethical standards and compliance with the Helsinki Declaration. The study was approved by the UCI Institutional Review Board (IRB; #20,194,936). Any modifications to the protocol are approved by the IRB via amendments.

**Fig. 1:**
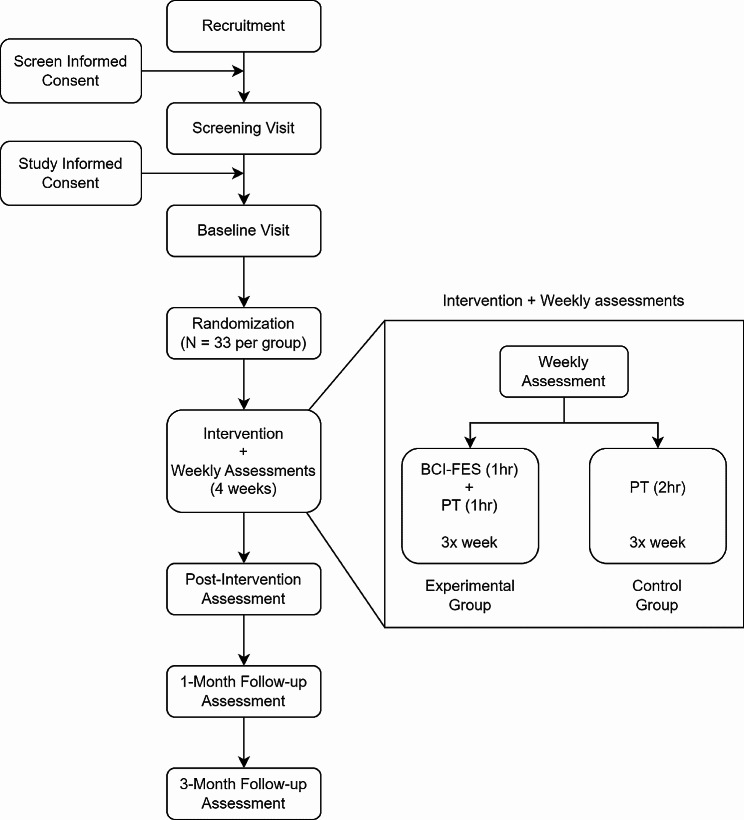
Overview of the trial

The entire study schedule outline, from screening and enrollment to the close-out, is represented in Fig. 1.

### Recruitment

Recruitment for this study commenced on January 15, 2020. In order to achieve our recruitment goals, we are employing the following strategies. An IRB-approved flyer is distributed at local stroke outreach events, professional meetings, stroke support groups, mass email/fax, social media, and paid advertisements. Additionally, a partial Health Insurance Portability and Accountability Act (HIPAA) waiver allows our clinical team to review the medical records of patients within the UCI Health system who have opted to be contacted for research purposes. The clinical team then identifies potentially eligible participants within this group.

Potential participants who express interest by contacting the researchers are briefed on the study’s details and invited for an initial screening visit (see Fig. 1). This visit includes assessments to determine their eligibility for the study. Participants sign the screening consent and medical release form prior to the visit. The study principal investigator (PI) then reviews their medical records to confirm the existence of a stroke diagnosis (based on the Magnetic Resonance Imaging: MRI scan), identify the time duration since the onset of stroke, and address other inclusion/exclusion criteria.

### Inclusion and exclusion criteria

#### Inclusion criteria

Inclusion criteria for the study are: (1) Age 18–80 years inclusively at time of consent, (2) Radiologically confirmed stroke, ischemic or intracerebral hemorrhage (ICH) in etiology, with day of onset at least 26 weeks prior to day of randomization, (3) Gait velocity *<* 0.8 m/s at screening and baseline visits, (4) FD in affected limb as defined by dorsiflexion active range of motion (AROM) via goniometry in seated position foot dangling is less than passive range of motion and less than 15°. (5) Plantarflexors spasticity *<* 3 on Modified Ashworth Scale (MAS), (6) Can walk *>* 10 m (with or without AFO, and cane or walker permitted) at a supervised level, (7) Can tolerate FES with pain no more than four on pain analog scale and has adequate muscle response of dorsiflexion ≥ 10°, (9) Passive Range of Motion ≥ 0°ankle dorsiflexion in subtalar neutral or with FES.

#### Exclusion criteria

Exclusion criteria for the study are: (1) A major, active, coexisting medical, neurological (apart from stroke) or psychiatric disease (apart from stroke), including alcoholism or dementia, orthopedic injuries, that substantially affects gait: As old orthopedic injuries may or may not affect gait, this exclusion related to orthopedic injuries can be waived at the discretion of the clinical team if the joint/muscles are back to normal motor and range of motion function, (2) A major medical disorder that substantially reduces the likelihood that a subject will be able to comply with all study procedures or safely complete study procedures. This includes, but is not limited to documented serious cardiac conditions, serious pulmonary conditions, legal blindness, end-stage renal or liver disease, or recent pulmonary embolism or deep venous thrombosis, (3) Resting systolic blood pressure ≥ 170 mmHg, diastolic blood pressure ≥ 100 mmHg at screening and baseline evaluations, (4) Implanted electronic device (e.g. pacemaker) or skull metallic implants (e.g. cranioplasty plate covering the leg motor area) with which study research procedures are contraindicated or incompatible, (5) Deficits in communication that interfere with reasonable study participation: language or attention impairment (score *>* 1 on National Institutes of Health Stroke Scale (NIHSS) items 9 and 11, respectively), (6) Significant cognitive impairment, defined as Montreal Cognitive Assessment score (MoCA) *<* 22: As MoCA scores for those with aphasia may be difficult to interpret, this exclusion criterion may be waived at the discretion of the clinical team, (7) A new symptomatic stroke apart from the index stroke occurred during the screening process and prior to randomization, (8) Life expectancy *<* 6 months, (9) Skin breakdown over electrical stimulation sites, (10) Received chemical denervation (e.g., Botox) to legs in the preceding 6 months, or expectation that chemical denervation will be administered to the leg prior to expected completion of the study, (11) Unable or unwilling to perform study procedures/therapy, or expectation of non-compliance with study procedures/therapy, (12) Pregnancy, (13) Significant pain (visual analog scale *>* 4), chest pain, or shortness of breath with walking, (14) Receiving any outside concurrent physical therapy involving the lower extremities at the time of enrollment or expectation that such therapy will be provided in the study up to one-month after study treatment, (15) Any general medical condition and psychosocial situation that substantially interferes with reasonable participate in study appointments, (16) Non-English speaking, such that subject does not speak sufficient English to comply with study procedures, (17) Concurrent enrollment in another investigational interventional study, (18) Severe depression, defined as Geriatric Depression Scale (GDS) Score *>* 11: As GDS scores for some patients may be difficult to interpret in the context of other neurological deficits (e.g., aphasia), this exclusion criterion may be waived at the discretion of the clinical team, (19) Concurrent use of FES orthosis for gait, (20) A new symptomatic stroke occurs apart from the index stroke during the screening process and prior to randomization.

If the participants meet the inclusion criteria and remain interested in continuing the study, they are scheduled for a baseline visit for additional assessments to reconfirm their eligibility. Following this, participants are informed about the specifics of the study by authorized personnel, including its protocol and the planned visits. Participants then sign a second informed consent form and are considered enrolled. They are then randomized into one of the two groups (experimental or control) and provided with a schedule for assessment (weekly and follow-up) and intervention visits.

### Randomization procedure and blinding

Random assignment of subjects to treatment arms (BCI-FES therapy with conventional PT vs. dose-matched conventional PT) is based on random permuted blocks using the SAS Proc Plan (SAS Institute, Cary, NC) routine with various random block sizes. Also, the randomization is stratified by baseline gait velocity (*<* or ≥ 0.4 m/s) or age (≤ or *>* 65 years old). After baseline assessments and enrolment, participant gait velocity and age data are entered in the randomization tables by the study coordinator to reveal the assigned group. The randomization tables are reviewed for every 5–10 subjects recruited to make sure the data are balanced. Outcome assessors are blinded to the assigned treatment group of the participants and do not have access to view specific treatment schedules, notes, or charts. Assessments are also performed in a separate building from where the treatment occurs. Unless a serious safety issue arises, treatment assignment will not be unblinded, otherwise, there are no circumstances under which unblinding is permitted.

### Assessments

Assessments are conducted at the following time points: at baseline, weekly during the intervention phase, immediately following the intervention. one-month postintervention and three-months post-intervention (Fig. 1). These assessments are designed to capture potential functional and neurophysiological changes associated with the interventions. Licensed clinical physical therapists and research personnel are recruited to perform assessments. The entire assessment team undergoes rigorous training with personnel who have extensive experience conducting research procedures involving EEG, EMG, and robot-based assessments. Additionally, assessment therapists complete thorough training in study clinical assessment procedures by becoming familiar with assessment equipment, completing online training, and certification of assessments for NIHSS, Modified Rankin Scale (mRS), and Lower-extremity motor Fugl-Meyer Assessment (FM-LE) every 12 months and the Montreal Cognitive Assessment every 2 years. The assessment team remains blinded to the participant’s intervention group allocation and randomization.

### Outcome measures and assessments

**Fig. 2:**
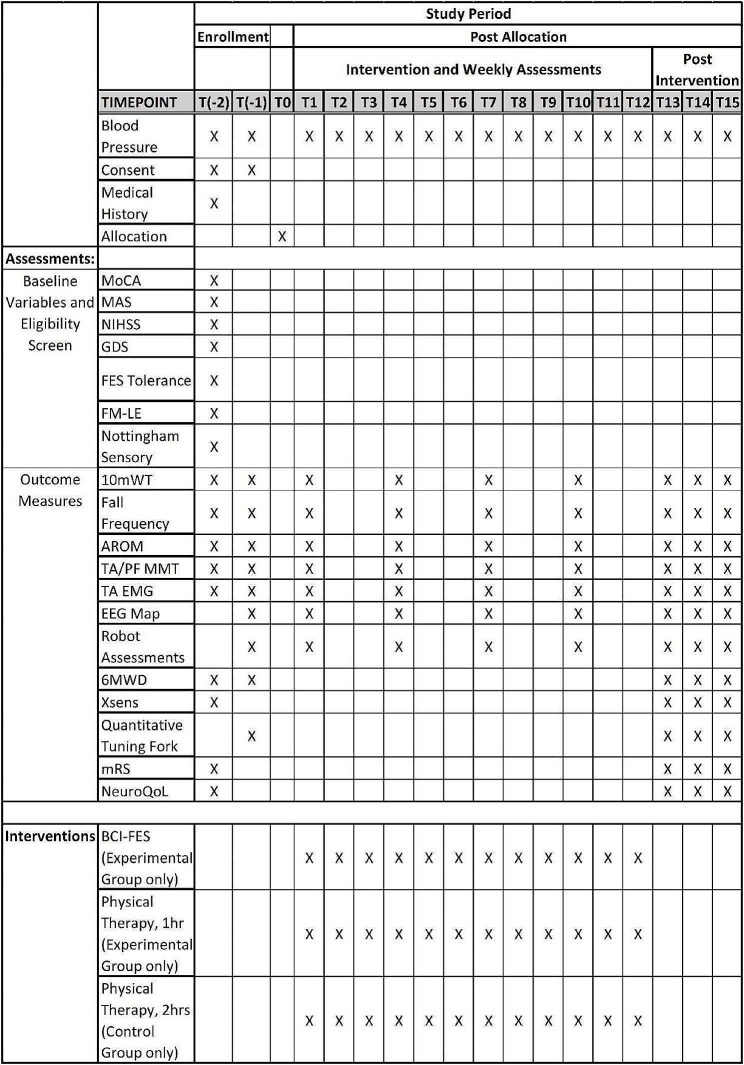
Standard Protocol Items: Recommendations for Interventional Trials (SPIRIT) figure. Timepoints- T(-2): Screening Assessment; T(-1): Baseline Assessment; T(0): Allocation; T(1, 4, 7, 10): Weekly Assessments; T(1–12): Intervention; T13: Post-Intervention; T14: 1-month Post-Intervention; T15: 3-months Post-Intervention

To quantify functional and neurological changes associated with the interventions and identify behavioral and physiological features predicting potential improvements, we have identified several outcome measures and corresponding assessment methods (see Fig. 2). For the primary outcome, we chose gait velocity, which is known to correlate highly with the degree of disability and social reintegration [17]. Secondary outcomes include:

1. Gait endurance.
2. Fall frequency.
3. Dorsiflexion AROM and strength.
4. EEG biomarkers.
5. TA volitional EMG.
6. Walking kinematics.
7. Sensory function.
8. mRS.
9. Neuro Quality of Life (NeuroQoL).
10. Robotic Assessments (AROM, Torque, Proprioception.

All clinical and functional assessments are conducted by a licensed physical therapist following standardized protocols.

Gait velocity is assessed by measuring the time to traverse the middle 6 m of a 10-meter walkway (10-meter walk test: 10mWT). The gait endurance is measured by calculating the distance traversed during a six-minute walk (6MWD). To additionally capture the subject’s walking pattern and kinematics for the secondary analyses, the Xsens Link system (Movella Technologies, Enschede, The Netherlands), which consists of wireless commercial inertial measurement units (IMUs), is strapped on the arms and legs during 6MWD. The dorsiflexion AROM is recorded manually using a goniometer. The dorsiflexion and plantarflexion muscle strength and function are tested via the manual muscle test (MMT). A quantitative tuning fork test is also included as a sensory measure to capture sensory function in the ankle. Outcome measures related to functional independence and quality of life are assessed using the mRS and NeuroQoL.

To provide a more objective assessment of the range of motion and strength, we designed a custom ankle robot, termed Ankle Measuring Proprioception Device (AMPD) [48]. AMPD uses sensors to record AROM and ankle torque and has motorized foot plates to employ novel proprioception assessments. These tests are necessary to quantify any proprioceptive changes accompanying the functional changes.

In our previous Phase I study [45], we identified changes in the EEG patterns over the sensorimotor region of the brain that were concomitant with gains in gait velocity and 6MWD. Thus, we include neurophysiological tests in the list of outcome measures to look for such changes in this trial. Specifically, EEG is recorded via a 64-channel cap (Mind Media USA Inc., California, USA, and ANT Neuro, Hengelo, Netherlands) during alternating periods of rest and dorsiflexion movements. For weekly assessments, we only use a subset of 25 electrodes concentrated around the leg motor area. For all assessment visits, we also record the TA muscle EMG underlying dorsiflexion.

In addition to assessing outcome measures, we include the following descriptor assessments at the screening visit to characterize each participant’s stroke-associated challenges and eligibility.

1. NIHSS.
2. MoCA.
3. GDS.
4. MAS.
5. Nottingham Sensory Score.
6. FM-LE.

We use the NIHSS and FM-LE score to quantify the baseline severity of stroke and leg motor impairment, respectively. We use the MAS to assess the presence and severity of spasticity and the Nottingham Sensory Score to assess sensory deficits at baseline. Cognition and symptoms of depression are assessed using MoCA and GDS, respectively.

### Interventions

#### BCI-FES

Subjects in the experimental group undergo placement of a 64-electrode EEG cap using standard techniques and a subset of eight channels, selected to capture brain areas subserving dorsiflexion (Cz, Cpz, C1, C2, Fz, FCz, FC1, FC2), are gelled and let to set for 15 min. Blunt needle scalp abrasion is then performed to reduce impedances to *<* 10 kΩ. The cap is connected to our custom BCI system and EEG signals are acquired at 200 Hz. This system represents a modification of our previous design [49], adapted to accommodate EEG signals. It has also been modified to include a custom-made FES module. The subjects are then instructed to alternate between 10 epochs of idling and 10 epochs of repeated dorsiflexion of their paretic foot. They are guided by textual/auditory cues, with each epoch lasting 10 s for a total of 200 s. Their EEG data are simultaneously collected and labeled by the corresponding state (“Idle” or “Dorsiflex”). Our custom software then analyzes the data to generate and calibrate a BCI decoder, which takes an additional 5–10 min. Subsequently, the subjects receive therapy in five minute online runs for a total of one hour. In each online run, they are guided by the same textual/auditory cues which prompt them to alternate between 15-second-long epochs of idling and repeated dorsiflexion of their paretic foot (a total of 20 epochs). In response, the BCI system decodes their underlying EEG in real-time and delivers FES to their TA muscle when Dorsiflex state is decoded. Accounting for short breaks, the expected number of online runs is 6–10 within a one-hour period. For a more detailed description of these procedures, the reader is referred to [45, 47].

#### Conventional PT

This intervention consists of a standardized regimen of activities typical of conventional post-stroke gait therapy, including passive/active range of motion exercises, lower-extremity muscle strengthening, and a progression of walking endurance and balance exercises.

The subjects in the experimental group receive one hour of BCI-FES therapy immediately followed by a one hour of conventional PT, as described above. A total of 12 such sessions are performed per subject at a rate of 3x/week over four weeks. The subjects in the experimental group receive two hours of conventional PT. A total of 12 sessions are performed per subject at a rate of 3x/week over four weeks.

In addition to the interventions, all participants (irrespective of the assigned group) are assigned a home exercise program by a commercially available home exercise software platform (PT Pal, Health Tech Pal Corp, Cherry Hill, NJ). This program is to be followed four times a week with compliance assessed via non-invasive sensors given to the subjects. For those without a mobile device, a pen-and-paper version of the home exercise program is also available. Subjects cannot undergo concurrent outside PT focused on the leg after enrollment until after one-month post-treatment. The amount of potential concurrent therapy is confirmed and recorded weekly during the assessment. A protocol violation form will be completed if the subject has participated in outside therapy for the leg.

### Participant retention, adherence to protocols, and withdrawal

Participation in the study is voluntary. The Protocol Rule Violation Forms will be used to document non-completion of scheduled therapy visits. Ideally, all subjects, irrespective of their assigned group, should be on a 4-week schedule to complete 12 sessions of treatment. However, to be considered ”on protocol”, subjects can be allowed up to five weeks to complete 12 sessions and cannot miss more than two of the 12 sessions. A session is considered complete at 75% percent of assigned minutes.

Other violations may include errors in randomization and performing evaluation visits outside of allowed periods. To ensure and promote participation retention, the study coordinator sends the entire schedule prior to the start of the study, and the participants plan around the provided schedule. Email reminders for all assessment times are sent one week before major assessment days (screening, baseline, and posttests). Physical therapists also remind the participants of the date and time of the next upcoming visit.

If a subject withdraws consent from the trial, the study team checks for any development of study-related adverse events. The subject is then requested to complete the End-of-Study form, including explaining why the subject is withdrawing consent.

### Adverse events and data safety monitoring

Adverse Events (AE) and Serious Adverse Events (SAE) are handled according to the guidelines provided by the National Institute on Aging (NIA) of the National Institutes of Health (NIH) [50]. Briefly, AE is defined as any untoward or unfavorable medical occurrence in a participant temporally associated with their involvement in the research, whether or not considered related to participation in the research. An AE is considered serious (SAE) if it is either life-threatening or is the cause of death, a new hospitalization, a new persistent and substantial disability, or the need for a new medical/surgical intervention to prevent these. The AEs will be classified according to severity (mild, moderate, severe), expectedness (expected, unexpected), and potential relatedness (definitely, possibly, not related) to the study intervention [50]. AEs and unanticipated problems are reported on the IRB website as per IRB protocol.

An independent Data and Safety Monitoring Board (DSMB) has been convened to assess the progress of the trial, the safety data, and critical efficacy endpoints. The members of the DSMB are experts in relevant fields such as neurology, rehabilitation technology, and biostatistics and with extensive experience in clinical trial methodology. The PI has appointed a DSMB Chair responsible for overseeing the meetings and developing the agenda in consultation with the PI. The members serve in an individual capacity and provide their expertise and recommendations and are independent of the study sponsor. A charter is maintained to define the roles and responsibilities of the DSMB, describe the data to be reviewed, and delineate the meeting process. The DSMB has access to the group-level statistics (experimental vs. control). The DSMB meets when one-third and two-thirds of the recruitment target is met to review the cumulative study data.

An unscheduled DSMB review can be triggered by an event in which a serious adverse and unanticipated problem arose that was deemed probably/definitely related to study procedures. The DSMB will be advised of the following stopping criteria: (1) if the proportion of subjects experiencing falls in those receiving BCI-FES therapy begins increasing significantly (i.e., *>* 10%) or if the fall rate among these subjects increases significantly from baseline; (2) More than 10% of subjects in the BCI-FES group experience a decrement in gait velocity of *>* 0.16 m/s.

All relevant clinical data are presented to the IRB annually, including reports of the DSMB along with serial measures gait velocity, dorsiflexion range of motion and torque, FM-LE, gait endurance test, and fall frequency. The investigative team is blinded to these, so they will be submitted directly from the DSMB to the IRB.

### Statistics

#### Primary analysis

The study design is a parallel randomized controlled trial with two arms: BCI-FES dorsiflexion therapy and dose-matched conventional PT. A total of 66 subjects will be randomized 1:1 into the two study arms. The primary outcome is gait velocity and the secondary outcomes are gait endurance, fall frequency, FM-LE, and dorsiflexion AROM and torque. Outcomes measures will be evaluated at baseline, weekly during therapy, immediately post-intervention, and one-month and threemonths post-therapy. The primary efficacy analysis is the intent-to-treat analysis of all subjects randomized. This will be based on a linear mixed model (LMM) with gait velocity measured at baseline and weeks one to four, with time, treatment group, and a group-by-time interaction factor used to assess the difference in the rates of change (improvement) in gait velocity between treatment groups during the period of active therapy. The model estimation will be based on restricted maximum likelihood (REML) with unstructured covariance among repeated measurements over time (using random intercept and slope terms).

### Secondary analyses

The LMM will also be applied to evaluate group differences for secondary outcomes and secondary endpoints at one-month and three-months post-therapy. Additional secondary/exploratory analyses will examine interactions between stroke features and response over time. The primary outcome is gait velocity immediately post-BCI-FES therapy, and the secondary outcomes are described above. To test whether specific stroke features (e.g., baseline gait velocity, dorsiflexion AROM, etc.) modify the response to treatment over treatment duration, an LMM model with three-way interaction (group, time, modifying factor) will be used to test whether the slope of the three-factor interaction is zero (i.e., to test where a specific stroke feature modifies the difference in treatment responses). Additional secondary analyses by subgroup (e.g., stratified by baseline gait velocity [*<* 0.4 m/s or ≥ 0.4 m/s] or age [≤ 65 or *>* 65 years old]), or analysis based on per-protocol population (PPP: all subjects without major protocol deviation) may be performed as appropriate.

If needed, analyses based on PPP for the primary and/or secondary outcomes will be considered. For this, the same LMM will be used, as described for the primary analysis above but restricted to the PPP. With respect to missing data, we note the LMM is a likelihood analysis [51], which is valid under a less restrictive assumption of data missing at random (MAR). In longitudinal clinical trials, it is preferred over simple methods of last observation carried forward, or complete case and available case analyses, which rely on the more restrictive assumption of data missing completely at random (MCAR). Sensitivity analysis will be based on multiple imputations [52].

### Power

The power analysis was based on an LMM with two treatment groups and five repeated measures (baseline and after weeks 1–4) with a significance level of 0.05. Previous studies have defined the clinically meaningful gait velocity improvement (minimal clinically important difference) in patients with post-acute stroke as being 0.16 m/s [53], including a multicenter randomized clinical trial that included conventional PT [54]. Thus, to ensure clinically meaningful effect size, we assume conventional PT will have an improvement of 0.25 m/s after four weeks of treatment. Data from prior studies and our preliminary data [45] suggest that the between-subject variance in gait velocity is 0.16 (m/s)^2^ and the within-subject correlation is 0.95. Table 1 below shows the power to detect a 30–40% improvement of BCI-FES over conventional therapy. Thus, the proposed study with *n* = 66 subjects (33 completed patients per treatment arm) has 83–87% power to detect an effect size/improvement of 35% for various between-subject variance (0.15 (m/s)^2^ to 0.17 (m/s)^2^) (which includes 10% attrition).

**Table 1:** Power to detect improvement in gait velocity due to BCI-FES over conventional therapy for *n* = 66 with various between-subject gait velocity variance

Effect size (Improvement)	Between-subject variance		
	0.15	0.16	0.17
30%	76.60%	73.90%	72.40%
**35%**	**87.50%**	**85.40%**	**83.20%**
40%	94.20%	92.80%	91.10%

### Data collection and management

Case report forms (CRF) for data collection for all assessment and intervention visits are maintained in an organized filing system for each subject. All de-identified hard copies are scanned and uploaded to the server. The treatment data and forms are accessible only to unblinded study members. The EEG, EMG, walking kinematics, and ankle robot data are stored electronically on a laptop that is backed up to a server. All CRF’s electronic data are identified only by a unique study ID number.

Authorized team members access the information for data entry in the UCI REDCap data management system. All data collected on CRFs are entered into the database using double data entry to ensure accuracy. REDCap also has built-in range checks for entered data values. The research team will not access data until this is required for safety monitoring by the DSMB, in which case the statistician will compile a report.

Additionally, all standard operating protocols for assessment and treatment methods are available electronically on a shared drive and as printed hard copies in binders in the assessment and treatment rooms. Study members, including assessment therapists, treatment therapists, and research technicians who administer the assessment/therapy, undergo extensive training to follow proper protocols and maintain consistency and standardization, ensuring the validity and reliability of the data collection.

### Study close out and dissemination plan

Study findings will be published in peer-reviewed scientific journals and presented at relevant conferences. Data will be available upon reasonable request from the primary author after publication. All relevant publications will be updated on the trial registry at the clinicaltrials.gov website.

## Discussion

Chronic gait impairments after stroke remain suboptimally addressed. Our early feasibility studies demonstrated that BCI-FES therapy for FD due to chronic stroke is safe and potentially effective [44, 45]. Therefore, this Phase II clinical trial is designed to formally evaluate the potential efficacy and safety of a novel neurorehabilitative technique to mitigate post-stroke gait impairments. Specifically, our primary objective is to determine whether BCI-FES therapy improves gait velocity more than dose-matched conventional PT. The result of this trial will determine if a future, large-scale, and definitive Phase III trial is warranted to establish the efficacy of BCI-FES therapy as a rehabilitation paradigm to improve gait velocity for chronic stroke patients. Future clinical trials for BCI-FES therapies and personalized stroke rehabilitation regimens may also benefit from this study and use it as a foundational guideline.

## Data Availability

No datasets were generated or analysed during the current study.
